# Complete Genome Sequence of an Enterohemorrhagic Escherichia coli O111:H8 Strain Recovered from a Large Outbreak in Japan Associated with Consumption of Raw Beef

**DOI:** 10.1128/MRA.00882-19

**Published:** 2019-10-03

**Authors:** Tsuyoshi Sekizuka, Kenichi Lee, Keiko Kimata, Junko Isobe, Makoto Kuroda, Sunao Iyoda, Makoto Ohnishi, Tetsutaro Sata, Masanori Watahiki

**Affiliations:** a Pathogen Genomics Center, National Institute of Infectious Diseases, Tokyo, Japan; b Department of Bacteriology I, National Institute of Infectious Diseases, Tokyo, Japan; c Department of Bacteriology, Toyama Institute of Health, Toyama, Japan; University of Arizona

## Abstract

We present the complete genome sequence of an enterohemorrhagic Escherichia coli O111:H8 strain. This strain was isolated from a hemolytic-uremic syndrome patient and was responsible for a large outbreak associated with the consumption of raw beef in 2011.

## ANNOUNCEMENT

Enterohemorrhagic Escherichia coli (EHEC) is a serious etiological agent of outbreaks of foodborne illness worldwide. More than 3,000 cases of EHEC infection are reported annually in Japan (1). In 2011, a large outbreak due to the consumption of raw beef was reported. A total of 181 patients, including 21 patients with acute encephalopathy (AE) and five deaths among 34 patients with hemolytic-uremic syndrome (HUS), were recorded (2, 3). EHEC O111:H8 and O157:H7 were isolated from the patients; however, the Shiga toxin 2a gene (*stx*_2_)-positive EHEC O111:H8 strain was the primary cause of the severe infection, according to serological testing of the patients (4). To reveal the characteristics of the strain, we determined its complete genome sequence, as the complete genome of the *stx*_2_-positive EHEC O111 strain has not been reported.

EHEC O111:H8 110512 was isolated as one of a few colonies grown on deoxycholate-hydrogen sulfide-lactose agar (Nissui Pharmaceutical Co., Ltd., Tokyo, Japan) from the fecal sample of a 6-year-old boy with HUS and AE. The strain was stored in 0.5% NaCl casein peptone medium (Kohjin Bio Co., Ltd., Saitama, Japan) until use. For DNA extraction, a single colony was inoculated into buffered peptone water (Nissui). The overnight culture was subjected to genomic DNA extraction by a DNeasy blood and tissue kit (Qiagen, Venlo, Netherlands). The genomic DNA libraries were prepared using a Nextera DNA sample prep kit (Epicentre Biotechnologies, Madison, WI, USA), followed by paired-end sequencing (2 × 150-mer) with a MiSeq instrument (Illumina, San Diego, CA). Additionally, the strain was sequenced on a PacBio RS II instrument (Pacific Biosciences, Menlo Park, CA). The genomic DNA was fragmented prior to PacBio sequencing using the Megaruptor (Diagenode, Denville, NJ). PacBio sequencing was performed by using the SMRTbell template prep kit 1.0 and polymerase binding kit P6 after size selection using BluePippin (Sage Science, Beverly, MA) with a cutoff value of 20 kb. In bioinformatic analysis, default parameters were used for all software. The complete genome of the strain was obtained by using both PacBio and Illumina reads. PacBio reads were assembled using the Hierarchical Genome Assembly Process version 3 in SMRT Analysis software (5). The minimum seed length for the assembly was 6,000. Short plasmids (≤10 kb) were assembled using A5 MiSeq software version 20140604 with Illumina short reads (6). The tentative circular contigs were subjected to error correction in three iterations using Pilon version 1.18 with default parameters and Illumina short reads (7). Annotation was performed in Prokka version 1.11 (8), InterPro version 49.0 (9), and NCBI BLASTP/BLASTX.

In Illumina and PacBio sequencing, the read numbers were 5,894,924 and 195,083 (mean read length, 7,798 bp), respectively. The total read lengths for Illumina and PacBio sequencing were 732,012,018 bp and 1,521,220,270 bp, respectively; the mean coverage was 132-fold and 273-fold, respectively. The complete genome sequence consists of a chromosome and seven plasmids as shown in Table 1. The strain carried a novel Stx2 prophage but did not carry a Stx1 prophage. These data would be helpful for the characterization of *stx*_2_-positive EHEC O111 and improve the resolution of the surveillance using whole-genome sequencing (10, 11).

**TABLE 1:** Statistics of the complete genome of the enterohemorrhagic Escherichia coli O111:H8 strain 110512

Statistic	Data for^*a*^:							
	Chromosome	pO111-110512_1	pO111-110512_2	pO111-110512_3	pO111-110512_4	pO111-110512_5	pO111-110512_6	pO111-110512_7
Size (kb)	5,302,257	118,827	78,470	46,350	6,795	6,674	5,423	1,546
No. of CDSs^*b*^	5,119	131	81	57	6	9	6	1
No. of rRNAs	22	0	0	0	0	0	0	0
No. of tRNAs	107	0	0	0	0	0	0	0
No. of prophages	13	0	0	0	0	0	0	0
GC content (%)	50.6	53.3	50.0	40.7	50.8	50.3	47.2	51.7
Chromosomal sequence type or plasmid Inc type	ST16	IncQ1, IncP	IncFII (pRSB107)	IncX1	ND	ND	ND	Col (MG828)
Antimicrobial resistance gene(s)	*bla*_EC-18_	*bla*_TEM-1B_, *strB*, *strA*, *sul2*	ND	ND	ND	ND	ND	ND
Accession no.	AP019761	AP019762	AP019763	AP019764	AP019765	AP019766	AP019767	AP019768

a ND, not detected.

b CDSs, coding DNA sequences.

### Data availability.

The complete genome sequence of EHEC O111:H8 strain 110512 was submitted to DDBJ/ENA/GenBank under the accession numbers AP019761 through AP019768. The primary data were deposited in the NCBI primary data archive, SRA, under the reference number DRP005302.
